# COVID-19 Infection-Related Thyrotoxic Hypokalemic Periodic Paralysis

**DOI:** 10.1155/2022/1382270

**Published:** 2022-08-24

**Authors:** Fadlila Fitriani, Vina Yanti Susanti, Mohammad Robikhul Ikhsan

**Affiliations:** ^1^Study Program of Specialty, Department of Internal Medicine, Dr. Sardjito General Hospital/ Faculty of Medicine, Public Health and Nursing, Universitas Gadjah Mada, Jl. Kesehatan No. 1 Sekip Sinduadi DIY, Yogyakarta 55284, Indonesia; ^2^Division of Endocrinology, Department of Internal Medicine, Dr. Sardjito General Hospital/ Faculty of Medicine, Public Health and Nursing, Universitas Gadjah Mada, Jl. Kesehatan No. 1 Sekip Sinduadi DIY, Yogyakarta 55284, Indonesia

## Abstract

SARS-CoV-2 infection induces the dysfunction of many organs including the thyroid gland through the role of ACE2 receptors as well as the consequences of the cytokine storm. Thyroid diseases such as subacute thyroidism, Graves' disease, thyrotoxicosis, and Hashimoto's thyroiditis have been documented in patients with SARS-CoV-2 infection. However, there are limited reports about the consequences of SARS-CoV-2 infection-related thyroid complications. We describe a case of man who was admitted to the emergency department due to repeated lower limb weakness since diagnosed with COVID-19. He had refractory hypokalemia and was treated with potassium replacement therapy for 2 months. However, the complaints continued. The patient has no history of thyroid disease, yet the laboratory result showed hyperthyroidism. Accordingly, he received oral thiamazole. As the laboratory parameters of the thyroid hormones improved, potassium levels returned to normal and the limb weakness stopped. This unusual thyroid complication should be considered in SARS-CoV-2 infection. The prompt diagnosis and appropriate therapy can reduce the burden of the disease.

## 1. Introduction

Coronavirus disease 2019 (COVID-19) caused by the severe acute respiratory syndrome coronavirus 2 (SARS-CoV-2) infection exhibits a broad range of clinical conditions, ranging from asymptomatic to severe organ damage [1]. The SARS-CoV-2 enters the human cell mediated by the angiotensin-converting enzyme 2 (ACE2) receptors which express in numerous organs, including the thyroid gland [2]. These interactions induce inflammation of follicular cells which alter both the structure and function of the thyroid gland [3]. Higher incidence of hyperthyroidism was reported in patients with COVID-19 infection, presumably due to immune response to the infection [4, 5]. Hyperthyroidism stimulates the Na-K ATPase pumps in skeletal muscles which in turn promotes the cellular K^+^ shifting, causing hypokalemia paralysis [6]. Thyroid function was shown to improve when the infection was resolved [7]. However, persistent hyperthyroid and hypokalemia were reported in case reports after COVID-19 infection [8, 9]. Thus, further understanding of SARS-CoV-2 infection-related thyrotoxic hypokalemic periodic paralysis is necessary to prevent the complications and determine the prognosis of these patients with COVID-19 infection.

## 2. Case Presentation

**Figure 1:**
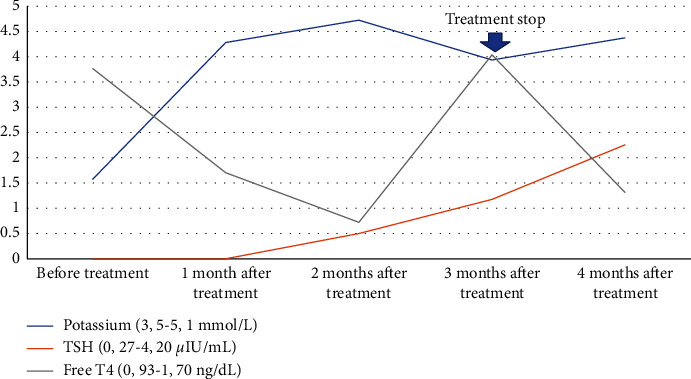
The changes of potassium, TSH, and free T4 before and after therapy of potassium replacement and thyroid hormone.

A 42-year-old male patient presented to the emergency department with weakness in his four extremities since a day before admission. The patient could stand but was too weak to walk. Tingling and pain sensations were occasionally reported in both the hands and the legs. He denied any trauma, paresthesia, or incontinence of the urinary or bowel systems. He has had cough and fever for the past five days without vomiting, diarrhea, or excessive sweating. Three months prior to admission, the patient had a COVID-19 infection. He experienced limb weakness and hypokalemia during hospitalization. He denied having any neck pain, and there was no swelling of the neck nor thyroid gland. He has no history of thyroid disease, hypertension, stroke, diabetes, nor malignancy. The patient had normal vital signs and underwent an electrocardiogram at the time of presentation. Crackles were found in both the lungs during a physical examination. There were no tumors or masses in his neck. A neurological examination indicated a bilateral upper and lower extremity motor impairment with similar strength of 4/5 and normal sensory function (Table 1). On laboratory finding, potassium level was 1.59 mmol/L, magnesium level was 2.23 mg/dL, free T4 (fT4) level was 3.75 ng/dL, and thyroid-stimulating hormone (TSH) level was <0.005 *µ*IU/mL. The potassium urine concentration was 5.05 mmol/L, with a potassium excretion fraction of 2.34%. Proteinuria was negative from urine analysis, and the renal function was normal (Table 2). Patient was diagnosed with thyrotoxic hypokalemic periodic paralysis. The patient received potassium chloride 50 mEq intravenously and oral thiamazole 5 mg twice a day for potassium correction. After potassium was restored to a normal level of 3.48 mmol/L, the extremities' weakness improved, with motor strength of 5/5 in all extremities. The patient was then discharged and advised to continue taking thiamazole 5 mg twice daily as well as oral potassium supplements twice daily. He had no weakness in his upper or lower extremities during the two-month follow-up, and his potassium, fT4, and TSH levels were normal. We stopped the medication and continue to give a monthly evaluation. The symptoms did not return and the thyroid hormone and potassium levels remained normal at the fourth month even without therapy (Figure 1).

## 3. Discussion

The SARS-CoV-2 infection has been linked to thyroid dysfunction through a complex interplay of hormonal and immunomodulatory signaling molecules [2]. ACE 2 receptors and transmembrane protein serine 2 (TMPRSS2), which are highly expressed in the thyroid gland, play a key role in virus entrance into the cells. The entry of the virus induces immune activation, producing proinflammatory cytokines and destroying the follicular cells of the thyroid [10]. Other mechanisms include indirect pathways caused by the virus's systemic inflammatory response, which causes thyroid gland destruction [2]. Subacute thyroiditis (SAT), a self-limited thyroidal disease usually induced by viruses, has been reported in several case reports of COVID-19 patients [11–13]. SAT is typically characterized by painful tender thyroid glands radiating to the ear, as well as systematic symptoms of fever, malaise, or anorexia. The disease usually consists of three phases, including thyrotoxic phase, hypothyroidism, and recovery into euthyroid over weeks to months. In a systematic review conducted by Trimboli et al., the median interval time for SAT development of patients with COVID-19 was 30 days [12]. However, the marked sign of a painful neck was absent in our patient during his COVID-19 infection course as well as in the months after the recovery. Muller et al. and Lania et al. reported cases of atypical thyroiditis without any complaint of neck pain [4, 14]. Patients with destructive thyroiditis are unlikely to have neck pain. This might be due to lymphopenia, which limits the inflammatory cell infiltration to the thyroid gland causing a lack of inflammatory reaction and capsular tension inside the thyroid [15]. Painless thyroiditis also occurs in autoimmune thyroid disease (AITD). The two major disorders of AITD are Graves' disease and Hashimoto's thyroiditis. Both diseases are characterized by the presence of specific antibodies such as TSH receptor antibody (TRAb), which is expressed in Graves' disease, and thyroglobulin autoantibody (Tg Ab) and thyroperoxidase autoantibody (TPO-Ab), which are expressed in Hashimoto's thyroiditis. Unfortunately, we did not conduct the antibody test on our patient.

The thyroid function alterations were observed in 50 COVID-19 patients with no history of thyroid disease. Low TSH and free T3 (fT3) were found in both COVID-19 and non-COVID-19 pneumonia. However, the declining levels of TSH and fT3 were positively correlated with the severity of pneumonia only in patients with COVID-19, thus suggesting the role of SARS-CoV infection in thyroid function alteration [16]. Another study conducted by Lania et al. reported a low level of TSH in 58 (20.2%) patients with COVID-19, with 31 of them exhibiting thyrotoxicosis from the laboratory findings. The study also discovered an inverse relationship between interleukin 6 (IL6) and TSH levels. However, a progressive decline of fT4 level without any drug intervention leads to the hypothesis of “silent” destructive thyroiditis [4]. In a postmortem study, an extensive apoptosis process in follicular epithelium which alters the follicular morphology was observed in the SARS-CoV-1 infection [17]. The apoptosis of the follicular cells induces the release of the preformed hormone, the elevation of fT4 concentration, and low TSH. In severely ill patients, such as seen in severe SARS-Co2 infection, the fT3 concentration was low due to the inhibition of deiodinase activity, thus producing T4 thyrotoxicosis, which has a less apparent clinical symptom of thyrotoxicosis [15, 18]. A high level of fT4 and a low concentration of TSH were observed after three months of SARS-CoV-2 infection in our patient. It was noted that low concentrations of immune cells such as CD4+, CD8+ T cells, B cells, and granulocyte cells were detected in peripheral blood of recovered plasma convalescence donors after two months of infection, thus indicating the long duration of immune disruption in patients with COVID-19 [19].

Thyrotoxic periodic paralysis (TPP) is a distinct manifestation of thyrotoxicosis where the patient develops a transient motor deficit due to acute hypokalemia. Due to the role of testosterone in the Na-K ATPase activity, TPP is more common in males, in line with our patient's gender [20]. One of the possible precipitating factors for TPP is infection. Kaushik et al. reported a case of hypokalemic paralysis associated with COVID-19 infection [21]. However, the thyroid function was normal, unlike in our case. Thyroid hormones promote the expression of Na-K ATPase in skeletal muscle cell membranes, allowing K+ to enter the intracellular compartment and causing hypokalemia in hyperthyroid patients in patients with COVID-19 has been reported in several case reports [22, 23]. The risk factors for hypokalemia in patients with COVID-19 include gastrointestinal loss, urinary loss, and diuretic use [22]. Furthermore, persistent hypokalemia was also reported in COVID-19 recovered patients [9]. The direct and indirect effects of SARS-CoV infection on the renal tubules influence the renin-angiotensin system (RAS). The binding of SARS-CoV-2 with the ACE2 receptors diminishes the ability of ACE2 to regulate RAS. Increasing RAS activity enhances the distal delivery of sodium and water to the collecting tubule of the kidney and the excretion of potassium, resulting in hypokalemia [9, 22, 23]. In our case, we excluded gastrointestinal loss since the patient had no symptoms of diarrhea or vomiting. The potassium urine excretion in our patient was normal, as well as the serum magnesium. Thus, we suggested hypokalemia in our case was due to the high level of thyroid hormones, leading to a thyrotoxicosis condition. In our case, intravenous potassium replacement therapy was effective in treating acute hypokalemic paralysis. The antithyroid drug and oral potassium supplementation were also effective in reducing the thyroid hormone, maintaining the serum potassium level, and inhibiting the recurrent TPP in our patient.

In conclusion, thyroid hormones play a critical role in regulating multiple organ systems. Close monitoring of thyroid hormones should be done in patients who exhibit the signs and symptoms of thyroid dysfunction, particularly in those with a history of thyroid disease and those receiving the antithyroid drug. Patients with COVID-related thyroid disease should be treated appropriately to prevent long-term complications which would affect their quality of life.

## Figures and Tables

**Table 1:** Demographic and clinical findings of the patient.

Demography	
Age	42 years
Sex	Male
Race	Asian, Indonesian
Residence	Urban

Clinical manifestation	
Symptoms	
Acute paralysis	Yes
Symptom of hyperthyroid	
Palpitation	No
Weight loss	No
Tremor	No
Heat intolerance	No
Physical sign	
Paralysis without sensory abnormality	Yes
Exophthalmos	No
Goitre	No
ECG finding	
Sinus tachycardia	No
Fibrillation	No

**Table 2:** Summary of the laboratory test.

Laboratory test	Patient level	Reference range
Total leucocyte count	12.40 × 10^3^/*µ*L	4.5–11.5
Hemoglobin	12.4 g/dL	13–18
Thrombocyte	414 × 10^3^/*µ*L	150–450
BUN	13.9 mg/dL	6–20
Creatinine	0.91 mg/dL	0.7–1.2
Sodium	146 mmol/L	136–145
Potassium	1.59 mmol/L	3.5–5.1
Magnesium	2.23 mg/dL	1.6–2.4
Calcium	2.25 mmol/L	2.15–2.55
Urine creatinine	56.56 mg/dL	39–259
Urine potassium	5.05 mmol/L	32–83
Potassium excretion fraction	2, 34%	—
fT4	3.75 ng/dL	1–1.7
TSH	<0.005 *µ*IU/mL	0.27–4.2
Urine routine test	Normal	

## Data Availability

The data used to present this case report are included within the article.
